# HOW NETWORKS POWERED MY CAREER STUDYING AGING IN AFRICAN AMERICANS

**DOI:** 10.1093/geroni/igz038.2231

**Published:** 2019-11-08

**Authors:** Keith E Whitfield

**Affiliations:** Wayne State University, Detroit, Michigan, United States

## Abstract

This presentation will provide some insights about aging in African Americans I have drawn from the network of scholars in my 30 year journey studying aging in African Americans. This journey has been focused on understanding biobehavioral interplays among demographic, genetic, familial, and psychosocial factors to understand individual variability in health and cognitive aging in African Americans. The presentation will include insights my mentors imparted to me, the contributions of my peers and what I learned from and tried to teach to my mentees. I will also share how my contributions were fueled by funding of my research and interactions with the staff at NIA. I will use data from my Carolina African American Twin Study of Aging, the Baltimore Study of Black Aging and the Study of Longevity and Stress in African Americans to provide examples. I will conclude with some suggestions for the next generation of scholars.

